# Impact of community-based health insurance on utilisation of preventive health services in rural Uganda: a propensity score matching approach

**DOI:** 10.1007/s10754-021-09294-6

**Published:** 2021-02-10

**Authors:** Emmanuel Nshakira-Rukundo, Essa Chanie Mussa, Nathan Nshakira, Nicolas Gerber, Joachim von Braun

**Affiliations:** 1 Center for Development Research (ZEF), University of Bonn, Genscherallee 3, 53117 Bonn, Germany; 2 Institute for Food and Resource Economics, University of Bonn, Nussallee 19, 53115 Bonn, Germany; 3 Department of Agriculture Economics, University of Gondar, Gondar, Ethiopia; 4 Kabale University, P.O. Box 317, Kabale, Uganda

**Keywords:** Community-based health insurance, Enrolment, Preventive health, Inverse probability weighting, Rural Uganda, I130, I150, I100

## Abstract

The effect of voluntary health insurance on preventive health has received limited research attention in developing countries, even when they suffer immensely from easily preventable illnesses. This paper surveys households in rural south-western Uganda, which are geographically serviced by a voluntary Community-based health insurance scheme, and applied propensity score matching to assess the effect of enrolment on using mosquito nets and deworming under-five children. We find that enrolment in the scheme increased the probability of using a mosquito net by 26% and deworming by 18%. We postulate that these findings are partly mediated by information diffusion and social networks, financial protection, which gives households the capacity to save and use service more, especially curative services that are delivered alongside preventive services. This paper provides more insight into the broader effects of health insurance in developing countries, beyond financial protection and utilisation of hospital-based services.

## Introduction

Community-based health insurance (CBHI) has emerged as credible pathway to universal health coverage in low income countries (Wang and Pielemeier 2012; WHO 2010). CBHI provides financial protection (Bonfrer et al. 2018; Habib et al. 2016; Nguyen et al. 2011, 2012) thereby enabling more access to curative health services (Browne et al. 2016; Jütting 2004; Mebratie et al. 2013; Ranson et al. 2007). However, developing countries suffer considerably from easily preventable illnesses such as malaria and diarrhoea. Malaria is estimated to have caused 228 million cases and over 400,000 deaths (WHO Global 2019). Eighty-five percent of cases were from only six countries in Africa and 94 percent of deaths were from Africa (WHO Global 2019). Meanwhile, diarrhoea episodes in 2016 were 4.5 billion resulting in over 1.6 million deaths in the same year (Troeger et al. 2018). Suffice to mention that the uptake of preventive health remains very poor in many low income countries (Dupas 2011) and instruments such as cash transfers, subsidies, vouchers, and waivers, have not been able to spur take up rates in expected margins. A relatively understudied intervention with regarding to nudging behaviour change for preventive health is voluntary health insurance.

Conventionally, an increase in health consumption after gaining health insurance is considered as moral hazard, especially when individual behaviour to risk aversion negatively changes. However, we anchor this study on the theoretical and philosophical distinction of the conventional understanding of moral hazard. We relate to the concepts of value of health (Einav and Finkelstein 2018) and of preferred or needed care (Grignon 2014) to suggest that an increase in health consumption after gaining health insurance is not always moral hazard. These concepts can be appropriately applied to low income countries where general health services availability and utilisation are low and the unmet need high. Improvements in health utilisation for such populations, therefore, might not be due to moral hazard but rather responding to the unmet need. In other words, opportunities for health utilisation simply did not exist, and health insurance makes them possible.

In this study, we use primary data from 464 households in rural south-western Uganda, some of whom were members of a large community based health insurance (CBHI) and others not. We then apply propensity score matching to account for observable selection into health insurance and estimate the effect of enrolment in insurance on using long-lasting insecticide treated mosquito nets (LLIN) and deworming children under 5 years. We find that CBHI increased the probability of LLIN usage by 26 percent and deworming by close to 18 percent. Respectively, this was equivalent to 84 percentage points and 29 percentage points of the control households’ usage rates.

These results are important in various dimensions. First, we demonstrate the effect of CBHI membership on preventive health care, an issue that has received limited research interest in developing countries, especially in Africa. A study most related to ours is Yilma et al. (2012) who study the effect of health insurance on LLIN in Ghana and find negative (and hence moral hazard) effect related with reduction in LLIN usage. We believe our results are different from Yilma et al. (2012) due to an integral part played by burial groups. In the case in Ghana, household enrolment is not based on group membership and previous insurance experience and yet in our case, these play a pivotal role. Moreover, these burial groups also have previous experience of preventive health information diffusion (Katabarwa et al. 2010, 2015). From a policy perspective, these results demonstrate that CBHI can be added to a range of interventions for preventive health. For Uganda, these results will also be pertinent in the efforts to promote CBHI within the planned national health insurance scheme, not only for financial protection and resource mobilisation purposes but also the overall effect on both curative and preventive health outcomes.

We map the rest of the paper as follows. In Sect. 2, through a laconic review of literature, we position this research in the theoretical underpinnings of understanding moral hazard in health insurance literature, especially making a distinction as to why improvement in health utilisation in our particular case might not count as moral hazard. In Sect. 3 we review developing countries’ literature on the link between health insurance and preventive health outcomes. Section 4 provides an overview of preventive health insurance in Uganda and gives a detailed view of the case study scheme, the data, and the identification strategy used. Section 5 provides descriptive and empirical results while Sect. 5 discusses possible pathways of impact and also points the reader to the some limitations of our analysis. Section 7 concludes. 2. 

## Health insurance, preventive health and moral hazard

In contextualizing the theoretical underpinnings in which health insurance might influence preventive health behaviour, we consider what a real effect of health insurance is and what the effect of moral hazard might be. Ex-ante moral hazard relates to the increase in risky behaviours and reduction in self-protection emanating from an insurance gain, for instance increasing smoking, or even reduction of preventive health care (Zweifel and Manning 2000). On the other hand ex-post moral hazard relates to the increase of care due to reduced costs of care. Ex-post moral hazard is therefore associated with “temptation consumption,” where individuals might consume “non-essential care” because it brings satisfaction. It is, therefore, important to underline that not all increased utilization of health care is moral hazard (Seog 2012). In general, preventive care is always excluded from the kind of care only taken for pleasure and satisfaction and hence has been foundationally excluded from moral hazard (Arrow 1963; Newhouse 2006; Pauly 1968). Moreover, more consumption of preventive health can lead to better current and future health status thereby lowering current and future premiums (Ellis and Manning, 2007).

However, there remains a necessity to distinguish between what is can be categorised as moral hazard and what is not. Einav and Finkelstein (2018) view moral hazard as the increase in healthcare spending emanating from higher health consumption by considering the concept of “value of care”. By value of care, Einav and Finkelstein (2018) imply how much future poor health (and associated costs) is curtailed by the higher spending on current care. For instance, preventive health would be considered high value care while emergency room visits for non-emergency conditions would be considered as low value care and hence increased costs for low value care considered broadly as moral hazard. If the costs of increased utilisation of preventive services (high-value care) are below the future costs of curative care in the event of no preventive efforts, current cost increases might not count as moral hazard. Brot-Goldberg et al. (2017) extend the concept of value of care by looking at “potentially valuable care” such as preventive health visits versus “potentially wasteful care” such as non-essential imaging services. It is therefore important to assess welfare gains from consumption of specific health services to clearly assess the extent of moral hazard (Baicker et al. 2015). This underlines the importance of estimating the estimated marginal productivity of an extra unit of preventive care in relation to the probability of illness (Zweifel and Manning 2000). Newhouse (2006) views these preventive services in the same light as drugs for the management of chronic illnesses such as diabetes or hypertension, for which there are no additional marginal costs for the consumption of an additional unit of healthcare. For these, he suggests that “…assuming there is no adverse effect on the use of other medical services, a subsidy that induces greater consumption of drugs should not even be considered moral hazard, so there is no trade-off between moral hazard and risk avoidance.” (Newhouse 2006).

Technically, assessing the welfare gains differs from intervention to intervention and in some, it is a rather complex procedure that requires a lot of data and a lot of time. It is not easy to distinguish between high value and low value care in establishing the welfare gains. However, one could make these two considerations to assess possible welfare gains and estimate the extent to which consumption of a given unit of health care or a health behaviour portrayed, might be moral hazard or not. The first is identifying whether the unit of health care consumed increases the risk of illness through reducing self-protection or whether it increases self-protection and hence leads to illness avoidance. In instances where the behaviour portrayed leans more to the latter than the former situation, a moral hazard-induced behaviour change or health consumption would suffice. Several empirical studies have shown this trend. Spenkuch (2012) found that health insurance in Mexico led to a reduction in the utilization of various preventive treatments, while Yilma et al. (2012) found a reduction in the use of insecticide-treated mosquito nets after enrolling for insurance in Ghana. Qin and Lu (2014) observed increases in smoking, heavy drinking, and consumption of high-calorie foods leading to obesity, in China. Stanciole (2008) observes similar changes in lifestyle behaviour in the United States after getting health insurance. On the opposite side, insurance induces consumption of preventive health services which result in more self-protection, better health, and a lower probability of illness (see Baicker et al. 2013; Finkelstein et al. 2012; Ghislandi et al. 2014; Marino et al. 2016; Simon et al. 2017). This further leads to reduced future health costs and a reduction in health disparities.

In a dimension slightly different from the value of care assessment, analysts can also test for the extent to which care is needed care or preferred care as per Grignon (2014). Elaborating on this concept, Grignon (2014) uses an example of body beautification plastic surgeries as preferred care while immunisations are needed care. Citing Evans (1983, 1984), Grignon et al (2018) suggest that utilised care is not always needed care and care foregone is not always care not needed. In the context of developing countries, where there is a very high burden of illness from preventable illnesses and where there is a high unmet need for preventive therapies (either from low knowledge and information or other access barriers and hence low utilization), establishing the extent of need and preference is important. Defining needed care should consider whether the care is essential for maintaining or improving health (in the sense of high value care Brot-Goldberg et al. 2017; Einav and Finkelstein 2018) and, in extreme definition, an evaluation of whether not receiving the care would lead to death, severe disability, or incapacity to live a healthy life. In this scenario, the financial (income effect as per Nyman (2001)) becomes only a secondary issue in assessing moral hazard. One example of moral hazard in this scenario might be, for instance, the preference for non-emergency caesarean section surgeries in delivery, often associated with health insurance coverage (Long et al. 2012). On the other hand, if losing insurance (either by completely losing coverage or by increasing prices) leads to less consumption of high value care (as Brot-Goldberg et al. (2017), Zhou et al. (2017) and Chandra et al. (2010) show in the United States), cost reduction from such efforts should not be celebrated as controlling moral hazard but rather a postponement of health costs to a future time when they will be more costly.

## Evidence from developing countries

Within low income countries, evidence of the impact on insurance on preventive health has been largely concentrated in Latin America, and with mixed findings. Giedion et al. (2010) found an eight percentage point increase in child immunisation and a 6% point increase in antenatal visits for mothers enrolled in the subsidised insurance program for the poor in Colombia. They also find that enrolment in the contributory health insurance was associated with increasing preventive dental check-ups by up to 45.6% points among the self-employed households. Still, in Colombia, Miller et al. (2013) found that utilisation of preventive physician visits increased by 29% points while the number of growth monitoring assessments increased by 1.5 times more among the poor. Other studies have also found effects regarding immunisation and growth monitoring (Bitrán et al. 2010; Cercone et al. 2010). Studies in Mexico, however, show mixed results. Insured adults were more likely to use preventive screening for hypertension, cholesterol, and cancer (Pagan et al. 2007; Rivera-Hernandez and Galarraga 2015), but other researchers do not find this evidence. King et al. (2009) find no effect on preventive health outcomes, while a closer examination of the same data by Spenkuch (2012) showed the presence of moral hazard with statistically and economically strong negative effects on several outcomes including taking a flu shot, a pap smear, mammogram and eye exam. In Africa, two studies in Ghana further provide a mixed picture. Gajate-Garrido and Ahiadeke (2015) finding improvements of up to 25% points more in anti-malarial medication for children among insured households. However, Yilma et al. (2012) reveal evidence of moral hazard with the reduction in the use of LLINs for insured households. Studies on a new health insurance scheme in Nigeria have indicated a sustained effect on blood pressure among CBHI participating households (Hendriks et al. 2016, 2014) though recent evidence suggests adverse selection in the decisions to enrol (Kramer 2017). By and large, there is still a dearth of evidence from low income countries to which this paper contributes to.

## Materials and methods

### Preventive health in Uganda

The provision of preventive health services in Uganda is synchronised with the current policy of free access to all health services in public health facilities (Nabyonga Orem et al. 2005, 2011). Services in private-not-for-profit (PNFP) health facilities are subsidised by government subsidies for primary health (Amone et al. 2005; Okwero et al. 2010), making a majority of preventive health services almost universally freely available (MOH 2013). Moreover, products such as LLINs are highly subsidised or provided for free through donor-supported programmes (USAID 2015). Since preventive services are available and subsidised, their utilisation should, in principle, be high. In fact, utilisation of such services is low. For instance, while LLIN ownership rates have increased substantially, close to 10 percent of households that own a net do not regularly use it (UBOS and ICF 2018), contributing to close to 16 million annual malaria infections (MOH 2016). Only 59% of the population had a hand washing facility and 26% had an improved sanitation facility (UBOS and ICF, 2018).

### The Kisiizi community-based health insurance scheme

The Kisiizi CBHI scheme started in 1996 (Musau 1999) and currently covers above 45,000 individuals in 220 groups (Kisiizi Hospital 2020). At the time of data collection, households paid annual premiums ranging from Uganda shillings equivalent to US$ 3 (Uganda shillings 11,000) per person for households of 8–11 members to US$ 8 (Uganda shillings 28,000) per individual in a two-person household with additional coverage for private wards. Kisiizi CBHI scheme is a rural scheme with no sophisticated method of controlling moral hazard and adverse selection. Instead, three conditions are applied at enrolment. First, households enrol as a unit, such that all members enrol at once. Partial enrolment is therefore not permitted. Secondly, enrolment is group-based. Households are organised in groups rather than individual household enrolment. However, this is not typical group insurance since there is no join liability within groups. Burial groups are, therefore, only used for information diffusion and collection of premiums. Conducting enrolment at household and group level has been found to control moral hazard and adverse selection in other CBHI schemes such as in Pakistan (Fischer et al. 2018). It is important to note that group leaders are not incentivised or punished by the scheme in undertaking these roles. Some groups have therefore experienced leadership challenges such as corruption and misuse of groups’ money, which has led to some of them dropping out of CBHI. These groups have different leadership styles, some electing leaders every couple of years while others haven’t elected leaders in a long time. The scheme does not have any influence of groups affairs since such groups always have other areas of operation (such as funeral support, village saving and lending, agricultural labour support etc.) that are beyond the scope of CBHI. Of the 210 groups registered in CBHI at the time of our data collection, over 95% of them were primarily burial insurance groups though with additional community social support function. Funeral insurance groups are central in the promotion of health insurance across other developing countries (Dercon et al. 2006, 2014). Membership in funeral groups is based on kin or neighbourhood relationships and, therefore exogenous. Virtually every household belongs in one, and in sometimes, non-membership attracted communal sanctions (Katabarwa 1999). There is, therefore, a very important social network dimension. Finally, the scheme employs a substantially long waiting period. Newly enrolled households typically wait for about 12 months to be fully covered, in which time they are required to pay 90%t of medical costs in the instance of hospitalisation. This waiting time is significantly longer than other schemes, such as one in Nigeria (Bonfrer et al. 2015).

**Fig. 1:**
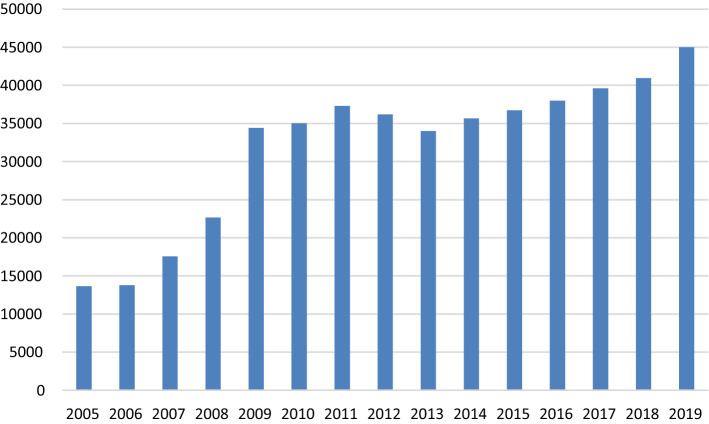
Coverage of the Kisiizi CBHI scheme. Source: Authors from scheme records

The scheme covers outpatient and inpatient services, surgeries and emergences services. Investigative and imagining procedures such as X-rays, ultra-sounds and laboratory investigations are also covered up to the full cost of the treatment. Elective surgical conditions are covered up to 50% of the cost. However, the insurance does not cover dental, optical procedures and what it considers as self-inflicted injuries such as those arising from alcohol consumption and substance abuse. Care for chronic illnesses and all other services sought from health providers outside the network of the scheme’s health facilities are also not covered. The total ceiling for each illness episode is about US$600. It is important to note here that the preventive health outcomes of interest here are provided for free by all health facilities in the country, under the public health financing policy that provides free health care at public health facilities and subsidises private health facilities with grants to provide essential care for free. Therefore, the effect of interest in this study is mainly a behaviour change effect for health utilisation rather than the income effect of health insurance (Fig. 1).

### The data

The scheme operates in 5 districts in south-western Uganda. However, we conducted our study in areas within a 15-km radius from the main health provision facility, the Kisiizi hospital. This area was comprised of 3 sub-districts (sub counties) in Kabale (now Rukiga) and Rukugiri districts, which have the highest concentration of insured households. According to the 2014 national census, these three sub-districts had a combined population of 105,600 people (UBOS 2014). We used a multi-stage simple random sampling criterion to select 464 households in fourteen (14) villages scattered in the three sub-districts in the scheme catchment area. We invited community leaders from the three sub-districts and conducted a village listing exercise, which produced 174 villages in total. Going by a criterion of (1) having a market, (2) a school or health centre, and (3) a road in the village, the leaders categorised the villages into rich and poor villages. We then listed 104 poor villages and 70 rich villages. Seven villages were then selected from each category using a raffle draw. In the selected villages, all households with a child between 6 and 59 months were selected. Village lists were carefully cleaned after double-checking with leaders and selected households. Altogether, 464 households were selected, and all responded to the survey conducted between August 2015 and November 2015.

The survey modules included a household demographic module, a child and maternal health module, and a nutrition module. Information on household social and economic welfare using durable assets holdings and other endowments in agriculture, water and sanitation, and housing was also collected to construct a wealth index, and social connectivity and perception modules were used to construct indices for social connectivity and perceptions. Village level information is also collected to account for village-level heterogeneity. The survey was administered on a computerised personal interviewing (CAPI) platform to enable cost efficiency in data transmission and avoid data losses (Caeyers et al. 2012).

Research ethical clearance was obtained through the University of Bonn Center for Development Research ethics committee. Ethical reviews were further conducted by the Mengo Hospital Research Ethics Review Committee, and the Uganda National Council of Science and Technology provided a research clearance certificate (SS-3936). Informed consent was acquired from all participants.

### Propensity score matching

Our identification strategy is guided by a theoretical model of preventive health, advanced by Dupas (2011). In this model of health investments, Dupas (2011) shows that health insurance acts as both as a curative and preventive health investment. As a curative investment, it provides cover for the financial shock due to illness in the current period. As a preventive investment, it reduces the probability of illness in future periods if it contributes to the utilisation of preventive services in previous and current periods.

To understand the relationships of interest, we apply propensity score matching (PSM), a robust quasi-experimental method that helps in accounting for possible endogeneity in differences between sub-samples exposed to the intervention and a sub-sample not exposed (Abadie and Imbens 2016; Jalan and Ravallion 2003; Smith and Todd 2005). The method is widely used in health evaluations, including those studying the effects of health insurance (Gustafsson-Wright et al. 2018; Trujillo et al. 2005; Woode 2017). With PSM, we are able to construct a control group that comprises of households that do not participate in CBHI but who have the same probability of participating based on a set on observable factors and compare them with those who participated in CBHI and estimate the effect of participation. PSM can reduce bias in observed differences between the treated and the control group if two conditions are met. The first is the conditional independence assumption or selection on observables assumption. For our case, this assumption requires that the determinants of participation in CBHI and those that determine the CBHI-related outcomes are observed. The second assumption is the common support or overlap assumption, which provides that the probability of participation for both treated and control groups should be similar between 0 and 1 ($0 < p\left( {T_{i} = 1{|}X_{i} } \right) < 1$). If these two conditions hold, then we can estimate the cross-sectional specification of the average treatment effect on the treated as follows.

$$ATET_{PSM} = E_{P\left( X \right)T = 1} \left\{ {E\left[ {Y^{T} | T = 1, P\left( X \right)} \right] - E\left[ {Y^{C} |T = 0, P\left( X \right)} \right]} \right\}$$

where ATET is the average treatment effect on the treated coefficient for outcome Y, which is either the use of an LLIN or a taking a deworming tablet in the previous six months, T denotes enrolment in CBHI while C denotes the control, not enrolled. P(X) is the probability of CBHI participation based on a vector of covariates X. To implement PSM, we use the Treatment Effects potential outcomes framework in Stata (Stata Corp 2015), implementing a PSM model with three nearest neighbours. We apply a calliper of 0.2 standard deviations of the propensity score, recommended by Austin (2011) and the standard errors are adjusted using the Abadie-Imbens method (Abadie and Imbens 2016). 

### Treatment, outcomes and covariates

In this study, the main treatment is membership in the Kisiizi CBHI scheme, which is given as a dummy that takes the value of 1 if the household was a member of the CBHI scheme and 0 otherwise. We estimate the probability of CBHI participation using a set of child, parent, household and village controls. The child-specific variables include age (in months), gender, birthweight, and exclusive breastfeeding for a full six months. We include parent control such as mother’s age, education status of the mother and father, and father’s employment status. We then include household controls including household size proportion of under-5 children in the household, household assets shown by total livestock units, an index of access to water and sanitation facilities, ownership of radio, ownership of a mobile phone, and whether a household was catholic or not. We then include various variables for household social connectivity, which influence the decision to enrol in CBHI. These include using mobile at least once in the last 30 days, having a neighbour in CBHI, membership in a farmer self-help group, and having a household member on a village leadership committee. We then include variables for household use of health services such as attendance of a postnatal clinic, attendance of antenatal care for the recommended four times, hospital treatment visit after sickness, and satisfaction with hospital waiting times. Finally, we include in the model five village-level variables that control for environment and variation at the village level.

### Covariate balancing and sensitivity

**Fig. 2:**
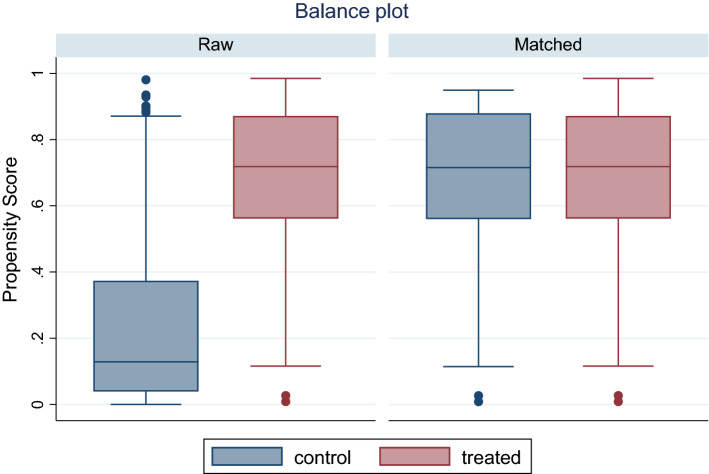
Balance box plots before and after matching

First, we provide results of covariate balancing after propensity score estimation. For our total usable sample of 455 households, all households have at least one nearest neighbour to provide a match. We provide results of balancing covariates in supplementary tables. Figure 2 below shows the box plot of raw and matched samples.

**Fig. 3:**
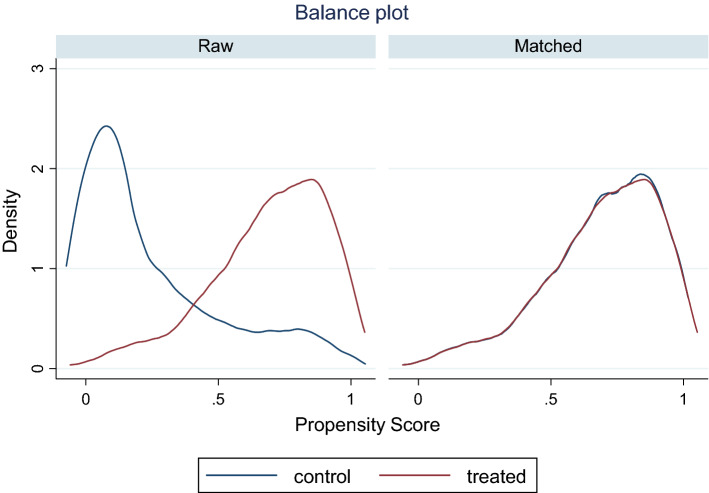
Kernel density plots for the distribution of the propensity score before and after matching

Figure 3 below shows the kernel density plots for the distribution of the propensity score before and after matching. By assessing both the box plots and kernel density plots, we are relatively comfortable of the balance achieved.

We further implement a more flexible PSM framework using PSMATCH2, the Stata user-written comment (Leuven and Sianesi 2003). Essentially, PSMATCH2 and our preferred implementation (Stata treatment effects) conduct identical analysis and results. However, PSMATCH2 allows us to conduct two more important procedures to test for robustness and sensitivity or our results. First, we narrow the calliper from 0.2 standard deviations of the propensity score (Austin 2011) to 0.015 standard deviations because narrow calliper width attain more precision (Lunt 2013). Secondly, the PSMATCH2 framework enables us to conduct additional sensitivity analysis for hidden bias by assessing Rosenbaum bounds to test for the level of unobserved heterogeneity (Becker and Caliendo 2007). We therefore show that our results are not sensitive to hidden bias at close to doubling the odds of assignment to treatment due to unobserved factors for LLIN or increase by over 50% for deworming.

## Results

### Descriptive results

We present mean differences in the CBHI and none CBHI households in Table 1 below. Households in CBHI were more likely catholic, employed in casual labour, belonged to farmers’ groups, more likely to possess a mobile phone, and were more likely to have attended four or more antenatal care visits. In addition, not surprisingly, CBHI participating households were more likely to have a neighbour in CBHI and live nearer to health facilities.

**Table 1:** Mean differences between CBHI and Non-CBHI households

	Mean no CBHI	Mean CBHI	Mean diff.	*t*-statistic
Child age − 6 to 11 months (59)	8.719	8.872	− 0.153	− 0.316
12–23 months (119)	17.210	18.263	− 1.053	− 1.567
24–35 months (118)	29.679	29.156	0.523	0.824
36–48 months (95)	41.398	42.010	− 0.612	− 0.774
< = 48 months (73)	54.381	54.259	0.122	0.133
Mother age (14–24 years) (126)	22.146	22.566	− 0.420	− 1.192
25–35 years (222)	29.362	29.510	− 0.148	− 0.385
> = 36 years (116)	39.851	40.789	− 0.939	− 1.236
Child is male^†^	0.487	0.473	0.014	0.292
Birth weight (kgs)	3.232	3.084	0.148	3.537***
Exclusive breastfeeding^†^	0.640	0.626	0.014	0.315
Religion is catholic^†^	0.383	0.660	− 0.277	− 6.143***
Father secondary education^†^	0.272	0.163	0.109	2.823***
Mother secondary education^†^	0.215	0.143	0.072	1.985**
Household < = 4^†^	0.391	0.414	− 0.023	− 0.500
Proportion of under-5	0.282	0.280	0.002	0.182
HDDS	4.027	4.187	− 0.160	− 1.366
Husband casual employment^†^	0.299	0.414	− 0.115	− 2.590***
Total livestock units	0.552	0.350	0.203	1.266
WASH index	0.013	− 0.017	0.030	0.245
Has a neighbor in CBHI^†^	0.521	0.911	− 0.390	− 9.927***
In a farmers’ group^†^	0.046	0.118	− 0.072	− 2.906***
Burial group size	80.100	60.138	19.962	8.844***
Has a radio^†^	0.644	0.657	− 0.013	− 0.291
Has a mobile phone^†^	0.685	0.792	− 0.107	− 2.599***
Used mobile money last 30 days^†^	0.433	0.468	− 0.035	− 0.752
Village leader (household member)^†^	0.157	0.182	− 0.025	− 0.718
Four or more ANC visits^†^	0.579	0.892	− 0.313	− 7.881***
Postnatal care visit^†^	0.812	0.847	− 0.035	− 0.990
Treated illness in last 14 days^†^	0.479	0.443	0.036	0.761
Satisfaction with waiting time^†^	0.644	0.571	0.072	1.585
Village has a health centre^†^	0.460	0.325	0.135	2.957***
Village has a traditional birth attendant^†^	0.747	0.759	-0.011	− 0.284
Village has a road^†^	0.943	0.882	0.061	2.349**
Log time (in minutes) to health facility	3.387	3.211	0.176	4.930***
Distance to health facility (kms)	4.489	3.374	1.114	4.150***
*Outcomes*				
Use of LLIN	0.356	0.552	− 0.195	− 4.278***
Child deworming	0.713	0.778	− 0.066	− 1.604
*N*	464			

T-statistics significance levels at *p < 0.1 for 10%, **p < 0.05 for 5% and, ***p < 0.01 for 1%. Observations for mothers’ age and wealth index per subgroup in parenthesis. ^†^denotes binary variables (1 = Yes, 0 = No)

However, households not in CBHI had healthier children, with a birth weight of about 200 g more. Both mothers and fathers in non CBHI households were more educated up to the secondary school level, and were generally located in better villages with either a school or a health centre. These households were also members of significantly larger burial groups. Burial groups are an essential informal social safety net (Dercon et al. 2006), and in larger groups, members might formally insure less due to wider risk-sharing networks to depend on (Genicot and Ray 2003). Regarding the outcomes, generally, CBHI households were more likely to use LLIN more and deworm children more, although a statistically significant difference is observed only with LLIN use.

### Empirical results

#### Determinants of enrolment

First, we show the results of the logistic regression of determinants of that enrolment in CBHI and report odds ratios in Table 2 below. Close to 44% of our sample were enrolled in CBHI. An increase in birth weight by 1 kg was associated with a 40 percent reduction in odds of participation in CBHI. Secondary school education for fathers was associated with a reduction in the odds of CBHI membership by 49.3%. This finding is somewhat intriguing because other studies have shown that higher education is associated in insurance enrolment (Dror et al. 2016). However, higher education is usually highly associated with rural out-migration (Bernard and Bell 2018; Schewel and Fransen 2018), implying that possibly, more educated husbands out-migrate. This, in turn, might disadvantage them in rural social networks such as burial group membership and participation which are necessary for this form of insurance. By extension, migration and education might relate to higher household incomes, which might induce people to opt for out of pocket payment for health services since they can afford it. We also observe that households in relatively larger burial groups are less likely to enrol in CBHI. An increase in burial group size by one household was associated with reducing the odds of CBHI enrolment by 3.8 percent. This speaks to the group size and risk-sharing behaviour (Genicot and Ray 2003). Finally, we observe that an increase in distance to a health facility by one kilometre reduces CBHI participation by 15.2%.

**Table 2:** Determinants of enrolment of CBHI

VARIABLES	Odds ratio	SE
Child age − 6 to 11 months (59)	–	(.)
12–23 months (119)	0.817	(0.350)
24–35 months (118)	0.855	(0.377)
36–48 months (95)	0.557	(0.266)
< = 48 months (73)	0.616	(0.324)
Child is male	1.022	(0.276)
Birth weight	0.400***	(0.129)
Exclusive breastfeeding	1.315	(0.382)
Mother age (14–24 years) (126)	–	(.)
25–35 years (222)	1.729	(0.596)
> = 36 years (116)	1.921	(0.764)
Religion is catholic	2.772***	(0.822)
Father secondary education	0.493**	(0.152)
Mother secondary education	0.558	(0.199)
Household < = 4	1.389	(0.493)
Proportion of under-5	0.720	(0.814)
HDDS	1.179	(0.148)
Husband casual employment	1.715*	(0.496)
Total livestock units	0.926	(0.082)
WASH index	0.996	(0.093)
Has a neighbor in CBHI	5.028***	(1.604)
In a farmers’ group	1.906	(0.934)
Burial group size	0.962***	(0.007)
Has a radio	1.008	(0.295)
Has a mobile phone	1.833*	(0.632)
Used mobile money last 30 days	1.314	(0.401)
Village leader (household member)	0.823	(0.313)
Four or more ANC visits	3.543***	(1.162)
Postnatal care visit	1.081	(0.440)
Treated illness in last 14 days	1.317	(0.363)
Satisfaction with hospital waiting time	1.162	(0.316)
Village has a health centre	2.178**	(0.840)
Village has a traditional birth attendant	1.199	(0.390)
Village has a road	0.560	(0.306)
Log time to health facility	0.152***	(0.080)
Distance to health facility	1.077	(0.068)
Constant	881.897***	(2,150.405)
Observations	455	

Robust standard errors in parentheses

^***^p < 0.01, **p < 0.05, *p < 0.1

Regarding factors that enhance the uptake of CBHI, we observe significant neighbourhood and information associations. Specifically, households with a neighbour in CBHI were 5 times more likely to enrol in CBHI themselves while owning a mobile phone increases the odds of enrolment by 1.8 times. We further observe that attending the recommended four or more antenatal care visits was also associated with 3.5 times more likelihood to enrolling while living in a village with a health facility also increased the odds of CBHI enrolment by over two times.

In Uganda, the current health policy stipulates that mandatory maternal health services and services at lower health facilities, including antenatal visits, are generally free of charge. This, therefore, points to possibly more health utilisation practices between insured and non-insured households.

#### Effect of CBHI on LLIN and child deworming

Proceeding to the main results, after controlling and balancing for a wide range of various observable covariates, we find that CBHI participation was associated with a 25.5% point increase in the probability of all household members using an LLIN. We further observe that the probability of child deworming increases by 17.5% points. In terms of potential outcomes for the non-CBHI households, these estimates correspond with 83.6 percent and 28.5% for LLIN and child deworming, respectively. Our results of sensitivity analysis using Rosenbaum Bounds (Becker and Caliendo 2007) show that for LLIN, our results are robust to sensitivity from unobserved bias of up to 1.85 critical values of gamma. while for child deworming, gamma critical values were 1.50. These sensitivity results imply that only at an 85% and a 50% increase in the odds of enrolment due to unobserved bias would our results for LLIN and deworming be sensitive to unobserved bias (Table 3).

**Table 3:** Average treatment effects

LLIN			Child deworming		
Coef. (se)	% of PO	Critical Value (Γ)	Coef. (se)	% of PO	Critical Value (Γ)
*ATT*					
0.255*** (0.0749)	83.6%	1.85	0.175** (0.0719)	28.5%	1.50
*Observations*					
455			455		

Abadie-Imbens Standard errors in parentheses

^***^p < 0.01, **p < 0.05, *p < 0.1

### Robustness checks with alternative estimators

To double-check the robustness of our results, we implement two additional strategies that are somewhat more conservative than our preferred strategy. We implement a closely related propensity score matching estimator but manipulate it further to achieve more precision at much lower calliper distances. We reduce thecalliper distances from 0.2 standard deviations to 0.015 standard deviations, pegging on the fact the reducing the calliper generally achieves more precision and less bias (Lunt 2013). Reducing the calliper essentially implements a more conservative cut-off on control and treated observations and results in a significant share of our sample being off support. Furthermore, we bootstrap the standard errors with 200 replications of the Abadie-Imbens Robust standard errors. From this strategy, the results presented in models 1 and 2 in Table, indicate that the point estimates for both LLIN and child deworming are closely similar.

Next, we implement a maxima-minima strategy to re-determine the area of common support (Caliendo and Kopenig 2008). In this strategy, we maintain observations whose propensity score lies between the largest propensity score in control and the smallest score in the treated units. In this case, we are able to remove observations with extreme propensity scores. We then implement our preferred strategy on this subsample. Results of this strategy shown in Models 3 and 4 of Table 4 show that though this strategy reduced point estimates by substantial margins, it still remained significant.

**Table 4:** Alternative estimation strategies for robustness

(1)	(2)	(3)	(4)	(5)	(6)
LLIN	Deworming	LLIN	Deworming	LLIN	Deworming
*ATT*					
0.266***	0.174*	0.219***	0.140**	0.274***	0.134*
(0.089)	(0.097)	(0.0755)	(0.0698)	(0.0771)	(0.0720)
*Observations*					
373	373	441	441	414	414

Abadie-Imbens robust standard errors in parentheses

^***^p < 0.01, **p < 0.05, *p < 0.1

Lastly we implement propensity score trimming which is advised in case of extreme propensity scores (Harder et al. 2010; Lee et al. 2011). While some analysts truncate the extreme weights to keep the sample (Harder et al. 2010), we effectively remove observation with extreme weights. We, therefore, remove 5% of the observations on each tail based on their propensity scores. This reduced our sample by 9%. Results from this strategy shown in Models 5 and 6 of Table 4 reveal a consistency of point estimates. We are therefore confident that using the alternative analytical strategies selected, some more conservative that our primary estimator, yields similar results.

## Discussion

We study the effect of CBHI on preventive health, in particular, sleeping under an LLIN and deworming children in households with under-5 children in rural south-western Uganda. Applying propensity score matching, we observe that enrolling in CBHI increased the probability of using a mosquito net by 26% and deworming children by 18%. It is important to note that despite high ownership of mosquito nets in rural Uganda, actual and consistent usage remains low (Ahmed and Zerihun 2010). The short term and long term usefulness of deworming cannot be understated results (Baird et al. 2016; Miguel and Kremer 2004). However, while the deworming medication is available for free for all under-5 children, only 61% of children in the country received the medication in 2016 (UBOS and ICF 2018). There is, therefore, a lot to do on changing behaviour in utilising preventive health medication.

### Impact pathways

A secondary question in this study is how these effects happen. To test the pathways of effects, we postulate three possible pathways of effect. We do not have sufficient data to fully prove all these pathways, but we make strongly suggestive analysis to show that the effects observed might be mediated through these channels.

The first one is the financial protection pathway emanating from savings, investments, and hence affordability of supplementary preventive health services that are often underutilised. While most of these preventive health services are publicly provided for free or at highly subsidised costs accessing them still comes at prohibitive costs. Lack of money was the main barrier to health services access for 44% of women in the country (UBOS and ICF 2018). Moreover, even with publicly-provided services, informal fees are common (Bouchard et al. 2012; Hunt 2010). Through financial protection, households in insurance are able to reduce the indirect financial barriers for accessing. In a previous study (Nshakira-Rukundo et al. 2020), we reveal that while household incomes were not associated with CBHI participation, each year of participation in CBHI was associated with close to 14 percent lower costs of care. We, therefore, think that through financial protection and associated savings, financial-related barriers of access to services are greatly reduced.

Our second pathway of effect is utilisation of health services. Throughout health insurance literature, financial protection is related to utilisation of health services. These are mostly curative services; however, in many instances, preventive services such as deworming, which is clinically administered, go hand in hand with access to curative services. In our models, we include a set of variables that control for health services utilisation. These are; (1) pregnant women attending antenatal clinics for at least the recommended four times, attending a postnatal clinic after delivery, treating an illness in the last 14 days, and level of satisfaction with health facility waiting times. To assess whether the mediation of curative health services is present, we exclude these variables from our main model and observe the coefficients. Results presented in Models 1 and 2 in Table 5 show that indeed, a significant portion of the effect is mediated through access to curative health services, most possibly enabled by insurance. Though the coefficients for LLIN and deworming remain significant, they reduce by 19.2% and 33.1%, respectively.

**Table 5:** Excluding information and health utilisation variables

(1)		(2)		(3)		(4)	
LLIN	Critical Value (Γ)	Deworming	Critical Value (Γ)	LLIN	Critical Value (Γ)	Deworming	Critical Value (Γ)
*ATT*							
0.205***	1.9	0.117*	1.9	0.228***	1.5	0.0809	1.5
(0.0754)		(0.0675)		(0.0621)		(0.0577)	
*Observations*							
455		455		458		458	

Abadie-Imbens robust standard errors in parentheses

^***^p < 0.01, **p < 0.05, *p < 0.1

The third possible pathway is information diffusion and social learning. Information diffusion might happen through prolonged exposure to behaviour change messaging (Behrman et al. 2004; Beshears et al. 2013) in such a manner that the longer the exposure period, the more learning and behaviour change. Knox (2018) used the length of exposure to health insurance as an instrument for health insurance enrolment in Mexico and found that enrolment increased demand for physical examinations and cancer screening. Moreover, intensive exposure can also lead to behaviour change and improve adoption rates of interventions (Kilian et al. 2016; MacIntyre et al. 2012). In our sample, we observed that burial groups in CBHI had 14 percent more meetings per month (2.4 meetings) compared to burial groups not in CBHI (2.1 times). Moreover, households can also belong to other voluntary social groups in addition to their funeral groups. Households in CBHI belonged in about 2.4 voluntary groups compared to 1.5 groups for households not in CBHI. With more group membership, we assume more information diffusion and social learning. The funeral group line of thought has been previously studied with findings suggesting significant impacts of funeral groups on preventive interventions (Katabarwa 1999; Katabarwa et al. 2010, 2015; Katabarwa et al. 2000a, b; Katabarwa et al. 2000a, b).

In the main model, we include a set of variables that control for information and social network in households. These include the size of a burial group a household belonged to, whether a household member was on the village leadership committee if a household member belonged to a farmers’ group if a household had a neighbour in CBHI, ownership of a radio, and of a mobile phone and using of mobile money at least once in the last 30 days. These variables are important in assessing information access in rural areas. To test if mediation through these variables is present, we exclude them from our main model and observe their contribution on the point estimates. Models 3 and 4 in Table 5 shows these results reavealing the importance of information. Not only do we observe an 11% and 54% downward shifting the point estimates for LLIN and deworming respectively, but the effect of deworming is fully mediated by information access. In additional, the critical values of gammy become even lower, further underlining the importance of information.

### Limitations of the study

This analysis applies the propensity score matching method to estimate the effect of insurance on two preventive health outcomes. While we find positive, statistically, and economically significant results, our analysis might have some limitations, some of which we would like to highlight. The first one is that generally, causal inference is best undertaken in an experimental setup, where selection into a treatment such as insurance, is completely random in such a manner that both treated and control units do not differ from anything else apart from the assignment. In the absence of random assignment, panel data and quasi-experimental set up can be used to efficiently mimic a random assignment. In our case, we neither have an experimental setup nor panel data to estimate the most efficient causal estimates. Matching only helps us to balance treatment and control groups on the observable covariates. However, there are a number of other unobserved factors that might influence both enrolment in CBHI and preventive health measures. Matching on cross-sectional data, therefore, comes in as a “second class” method for causal inference. While our results are helpful in understanding how CBHI can nudge behaviour change towards better preventive health, there is a need for more studies in this regard, especially those that use more robust methods of causal analysis.

Secondly, our data also lack critical details that would help in precisely assessing certain things. For instance, we do not have actual data on the number of visits to a health facility for curative services though we know if a household visited a health facility or not. We also did not precisely measure information on health insurance. Though this particular insurance is largely spread through traditional networks of burial societies, having more information and knowledge increases the propensity to enrol in insurance. We, therefore, use only proxies that are able to suggestively and not conclusively tell us what we see here. The next efforts of research would learn from this process to design more detailed research tools to conclusively test certain hypotheses.

## Conclusion

This study contributes to the limited evidence on the effect of health insurance on preventive health in developing countries. The study applied propensity score matching on data from rural south-western Ugandan households and showed that CBHI enrolment increased the probability of using long-lasting mosquito nets by 26% (84% points of the control group) and deworming by 18% (29% points). The limitations of propensity sore matching notwithstanding, we believe this paper makes credible contributions to health insurance effects beyond utilisation of curative health services and financial protection. This study is of critical interest to Uganda policymakers, especially those currently involved in the process of introducing a national health insurance scheme.

## Appendix

See Tables

**Table 6:** Covariate balance in *teffects psmatch*

	Standardized differences		Variance ratio	
	Raw	Matched	Raw	Matched
Child age (base: 6–11 months)				
12–23 months	− 0.0379983	0.119652	0.959846	1.171875
24–35 months	0.09097	− 0.0259	1.109266	0.974992
36–48 months	− 0.0860386	− 0.08294	0.880722	0.883462
49–60 months	− 0.0895301	− 0.05589	0.844194	0.895833
Mother age (base: 14–25 years)				
25–34 years	0.0387743	− 0.18073	1.004158	1.025862
35 – max years	− 0.0461867	0.225658	0.947574	1.436412
Birthweight	− 0.3305164	− 0.14417	0.945516	1.336844
Exclusive breastfeeding	− 0.0397104	0.084886	1.022706	0.966113
Household < = 4	0.048579	− 0.00337	1.019497	0.998918
Proportion of U-5	− 0.0161735	0.120928	0.630676	0.937607
Child is male	− 0.0146868	0.086911	0.999827	1.016486
Father education (1 = secondary)	− 0.2750445	0.18122	0.686507	1.486959
Mother education (1 = secondary)	− 0.1937203	− 0.02794	0.724176	0.946555
Household diet diversity score	0.1384825	0.072777	1.167653	0.973849
Husband employment—Casual	0.2179859	0.081873	1.140562	1.037752
Total livestock units	− 0.1331961	0.149273	0.112577	1.634891
Has a radio	0.016624	0.058936	0.990569	0.965889
Catholic	0.5702879	− 0.12174	0.959804	1.101258
Water and sanitation index	− 0.0289517	0.17497	0.795295	1.256359
Has a neighbour in CBHI	0.9704995	− 0.20274	0.312018	2.201872
Burial group size	− 0.8279865	0.1254	0.756651	0.906686
Attended < = 4 ANC visits	0.7554217	− 0.07405	0.387321	1.230218
Attended a postnatal visit	0.0880367	0.138308	0.85808	0.794122
Treatment in last 14 days	− 0.0804218	0.148962	0.991454	1.054138
Satisfaction with hospital waiting times	− 0.1396651	0.046941	1.065914	0.98805
Has a mobile phone	0.2200017	0.140483	0.782221	0.841705
Used mobile money in last 30 days	0.0617153	0.120792	1.012443	1.029764
Household member village leader	0.0510106	− 0.13952	1.095064	0.817024
Belongs to a farmer's group	0.2653271	0.097734	2.357367	1.289377
Village has a health center	− 0.2841258	0.311707	0.883355	1.425439
Village has a traditional birth attendant	0.0254586	− 0.08401	0.97132	1.11919
Village has a road	− 0.2314454	0.005101	2.037369	0.988173
Time to hospital (log)	− 0.463757	− 0.01049	1.607905	1.236815
Distance to hospital (kms)	− 0.4028906	− 0.0936	0.221273	0.762993

^6^ and

**Table 7:** Covariate balancing in *Psmatch2*

	Unmatched	Means		Bias reduction		*t*-test	
Variable	Matched	Treated	Control	%bias	% reduct bias	t	*p* > t
*Child age (base: 6–11 months)*							
12–23 months	U	0.250	0.267	− 3.8		− 0.4	0.688
	M	0.250	0.200	11.4	− 200	1.2	0.232
24–35 months	U	0.275	0.235	9.1		0.97	0.334
	M	0.275	0.287	− 2.7	70.6	− 0.26	0.796
36–48 months	U	0.185	0.220	− 8.6		− 0.91	0.365
	M	0.185	0.218	− 8.3	3.7	− 0.83	0.407
49–60 months	U	0.140	0.173	− 9		− 0.94	0.346
	M	0.140	0.160	− 5.5	38.6	− 0.56	0.577
Child is male	U	0.475	0.482	− 1.5		− 0.16	0.877
	M	0.475	0.432	8.7	− 489.3	0.87	0.385
Birthweight	U	− 0.081	0.066	− 33.1		− 3.49	0.001
	M	− 0.081	− 0.022	− 13.3	59.8	− 1.44	0.150
Exclusive breastfeeding	U	0.620	0.639	− 4		− 0.42	0.674
Mother age (base: 14–25 years)	M	0.620	0.578	8.6	− 116.8	0.85	0.396
25–34 years	U	0.490	0.471	3.9		0.41	0.682
	M	0.490	0.580	− 18	− 363.6	− 1.81	0.071
35 – max years	U	0.235	0.255	− 4.6		− 0.49	0.626
	M	0.235	0.147	20.5	− 343.8	2.26	0.025
catholic	U	0.655	0.380	57		6.03	0.000
	M	0.655	0.712	− 11.8	79.4	− 1.22	0.224
Father education (1 = secondary)	U	0.165	0.278	− 27.5		− 2.88	0.004
	M	0.165	0.103	15	45.6	1.81	0.071
Mother education (1 = secondary)	U	0.145	0.220	− 19.4		− 2.03	0.043
	M	0.145	0.155	− 2.6	86.6	− 0.28	0.780
Household < 4 members	U	0.420	0.396	4.9		0.51	0.607
	M	0.420	0.422	− 0.3	93	− 0.03	0.973
Proportion of U-5	U	0.281	0.283	− 1.6		− 0.17	0.866
	M	0.281	0.266	10.8	− 568.4	1.21	0.227
HDDS	U	4.205	4.032	13.8		1.47	0.141
	M	4.205	4.110	7.6	45.1	0.73	0.467
Husband employment—Casual	U	0.410	0.306	21.8		2.32	0.021
	M	0.410	0.370	8.4	61.6	0.82	0.413
Total livestock units	U	0.346	0.565	− 13.3		− 1.35	0.179
	M	0.346	0.246	6	54.7	1.49	0.136
Water and sanitation index	U	− 0.007	0.031	− 2.9		− 0.3	0.761
	M	− 0.007	− 0.212	15.6	− 439.1	1.75	0.081
Has a neighbour in CBHI	U	0.915	0.522	97		9.96	0.000
	M	0.915	0.963	− 11.9	87.7	− 2.03	0.043
Belongs to a farmer's group	U	0.120	0.047	26.5		2.88	0.004
	M	0.120	0.090	10.9	58.9	0.98	0.329
Burial group size	U	60.175	80.063	− 82.8		− 8.69	0.000
	M	60.175	57.308	11.9	85.6	1.25	0.211
Has a radio	U	0.655	0.647	1.7		0.18	0.860
	M	0.655	0.627	5.9	− 256.8	0.59	0.556
Has a mobile phone	U	0.790	0.694	22		2.31	0.021
	M	0.790	0.730	13.8	37.4	1.4	0.161
Used mobile money in last 30 days	U	0.470	0.439	6.2		0.65	0.514
	M	0.470	0.410	12	− 94.9	1.21	0.228
Household member village leader	U	0.180	0.161	5.1		0.54	0.588
	M	0.180	0.237	− 15	− 194.9	− 1.4	0.164
Attended < = 4 ANC visits	U	0.895	0.584	75.5		7.79	0.999
	M	0.895	0.917	− 5.3	93	− 0.74	0.459
Attended a postnatal visit	U	0.845	0.812	8.8		0.93	0.354
	M	0.845	0.792	14.1	− 60.5	1.38	0.167
Treatment in 14 days	U	0.450	0.490	− 8		− 0.85	0.395
	M	0.450	0.377	14.7	− 82.4	1.49	0.137
Satisfaction with hospital waiting times	U	0.575	0.643	− 14		− 1.48	0.139
	M	0.575	0.552	4.8	65.8	0.47	0.639
Village has a health center	U	0.325	0.463	− 28.4		− 3	0.003
	M	0.325	0.190	27.8	2	3.12	0.002
Village has a traditional birth attendant	U	0.760	0.749	2.5		0.27	0.788
	M	0.760	0.795	− 8.1	− 218.7	− 0.84	0.401
Village has a road	U	0.880	0.945	− 23.1		− 2.5	0.013
	M	0.880	0.878	0.6	97.4	0.05	0.959
Time to hospital (log)	U	3.208	3.387	− 46.4		− 4.98	0.000
	M	3.208	3.212	− 1.1	97.6	− 0.1	0.916
Distance to hospital (kms)	U	3.370	4.484	− 40.3		− 4.11	0.000
	M	3.370	3.538	− 6.1	85	− 0.94	0.35

^7^
